# Supplementary material for: A fungal NRPS-PKS enzyme catalyses the formation of the flavonoid naringenin
Source: Nat Commun. 2022 Oct 26;13:6361. doi: 10.1038/s41467-022-34150-7 (PMC9606254; doi:10.1038/s41467-022-34150-7)
Supplement: Supplementary file 1 — Supplementary Information [file 41467_2022_34150_MOESM1_ESM.pdf]

A fungal NRPS-PKS enzyme catalyses the formation of the flavonoid naringenin

Zhang *et al*.

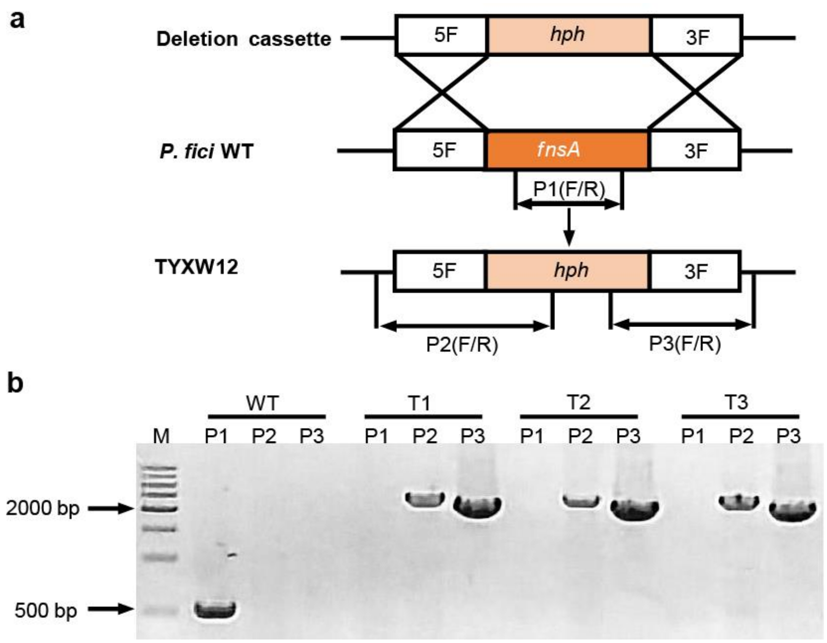

Supplementary Figure 1. Gene disruption of *fnsA* (*PFICI_04360*) in *P. fici*. a, Scheme illustration of *fnsA* disruption in *P. fici*. P1(F/R), P2(F/R) and P3(F/R) represent the primers used for mutant selection. **b**, Confirmation of the *fnsA*-knockout strains by diagnostic PCR. The specific bands (about 2.5 kb and 2.0 kb) were detected in mutants (T1-T3) using the P2(F/R) and P3(F/R) primers but not in WT. *fnsA* was detected in WT using P1(F/R) primers, but not in the mutant strains (T1-T3). T, transformants. Each experiment (**b**) was repeated twice independently with similar results. Source data are provided as a Source Data file.

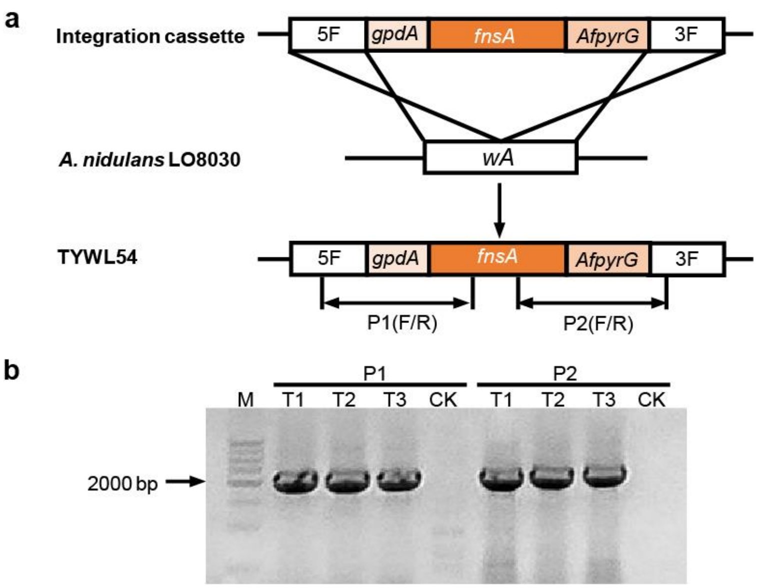

Supplementary Figure 2. Heterologous expression of *fnsA* (*PFICI_04360*) in *A. nidulans*. a, Scheme illustration of *fnsA* heterologous expression in *A. nidulans*. P1(F/R) and P2(F/R) represent the primers used for mutant selection. **b**, Confirmation of *A. nidulans* expressing *fnsA* strain by diagnostic PCR. The specific bands (about 2.0 kb) were detected in mutants (T1-T3) using the P1(F/R) and P2(F/R) primers but not in CK. T, transformants. Each experiment (**b**) was repeated twice independently with similar results. Source data are provided as a Source Data file.

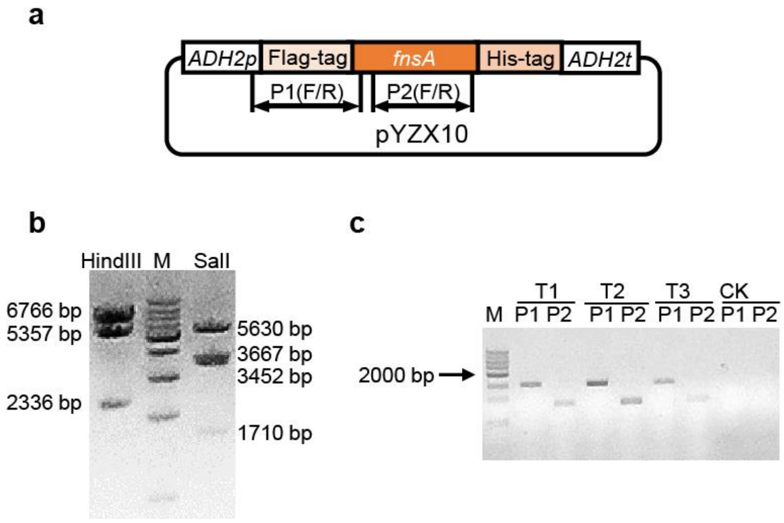

Supplementary Figure 3. Heterologous expression of *fnsA* (*PFICI_04360*) in *S. cerevisiae*. a, Scheme illustration of *fnsA* heterologous expression vector (pYZX10) in *S. cerevisiae*. **b**, Confirmation of the vector pYZX10 by enzyme digestion. **c**, Confirmation of *S. cerevisiae* expressing *fnsA* by diagnostic PCR. The specific bands (about 1.0 kb) were detected in mutants (T1-T3) using the P1(F/R) primers but not in CK. T, transformants. Each experiment (**b**) was repeated twice independently with similar results. Source data are provided as a Source Data file.

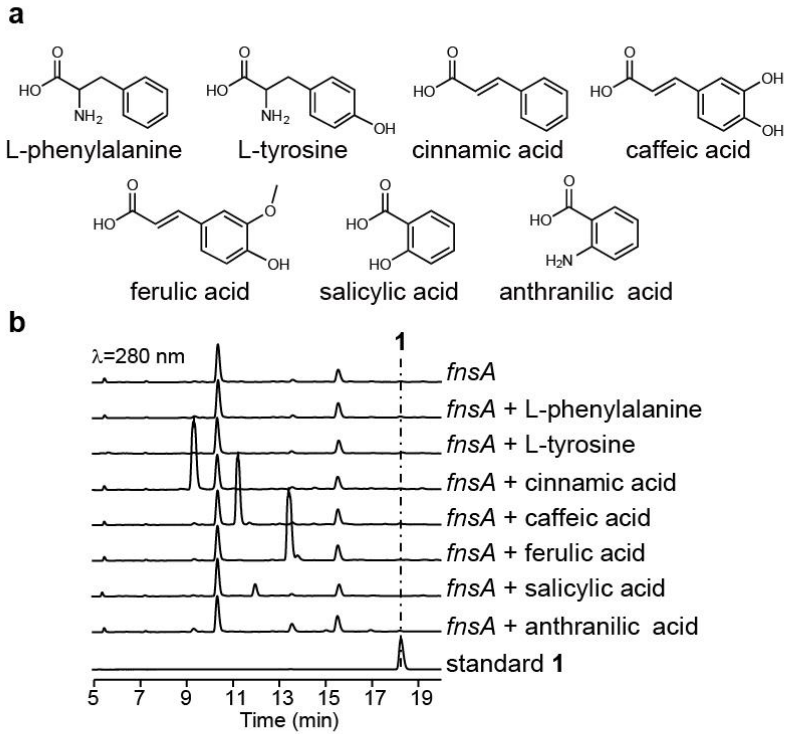

Supplementary Figure 4. HPLC analysis of secondary metabolites of *S. cerevisiae* carrying *fnsA* by feeding with different substrates. a, Structures of the feeding substrates, except for *p*-coumaric acid and *p*-hydroxybenzoic acid. **b**, HPLC analysis of secondary metabolites from feeding experiments in *S. cerevisiae* carrying *fnsA*. Substrates were supplemented at a final concentration of 0.5 mM. No target product was detected. **1**, naringenin. Source data are provided as a Source Data file.

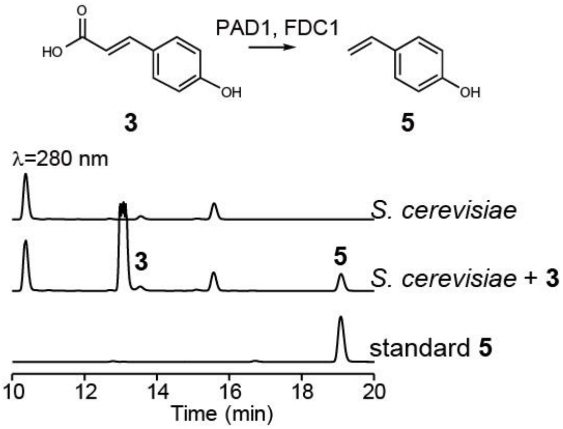

Supplementary Figure 5. HPLC analysis of secondary metabolite of *S. cerevisiae* feeding substrate 3. 3 was decarboxylated into **5** with the catalysis of endogenic genes *PAD1* (phenylacrylic acid decarboxylase gene) and *FDC1* (ferulic acid decarboxylase gene) in *S. cerevisiae*[1]. **3** was added to a final concentration of 0.5 mM into *S. cerevisiae*. **3**, *p*-coumaric acid; **5**, *p*-hydroxystyrene. Source data are provided as a Source Data file.

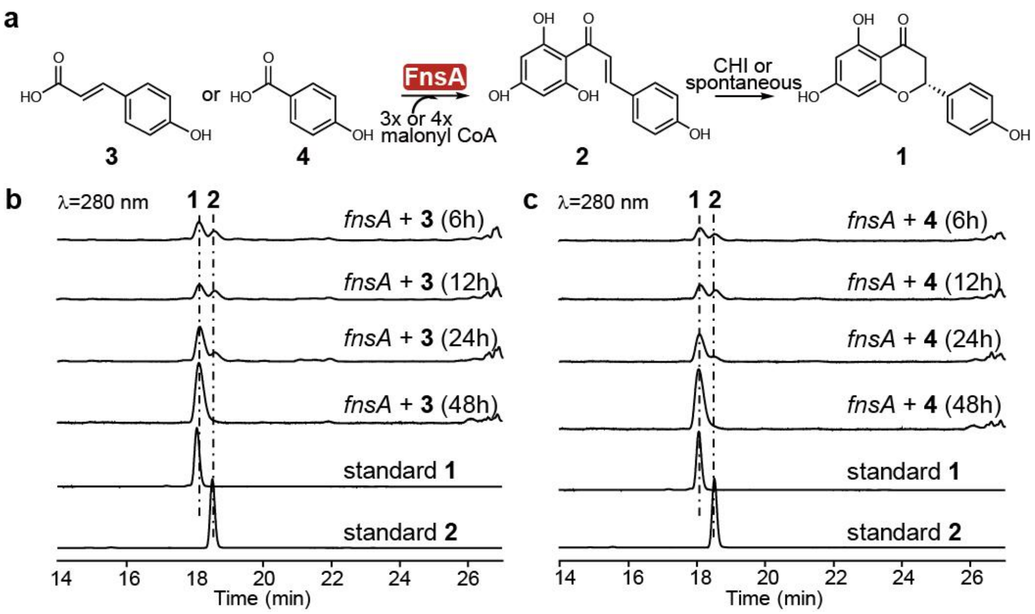

Supplementary Figure 6. HPLC analysis of secondary metabolites of *S. cerevisiae* carrying *fnsA* by feeding experiments. **a**, Schematic illustration of the function of FnsA. **b** and **c**, HPLC analysis of secondary metabolites in *S. cerevisiae* containing *fnsA* when feeding with **3** and **4** during different time-course**. 2** was isomerized into **1** in *S. cerevisiae* by non-enzymatic reaction or with the catalysis of an endogenous chalcone isomerase (CHI) when feeding with **3** or **4. 2** completely converted into **1** after 48 h. Precursor was added to a final concentration of 0.5 mM. **1**, naringenin; **2**, naringenin chalcone; **3**, *p*-coumaric acid; **4**, *p*-hydroxybenzoic acid.

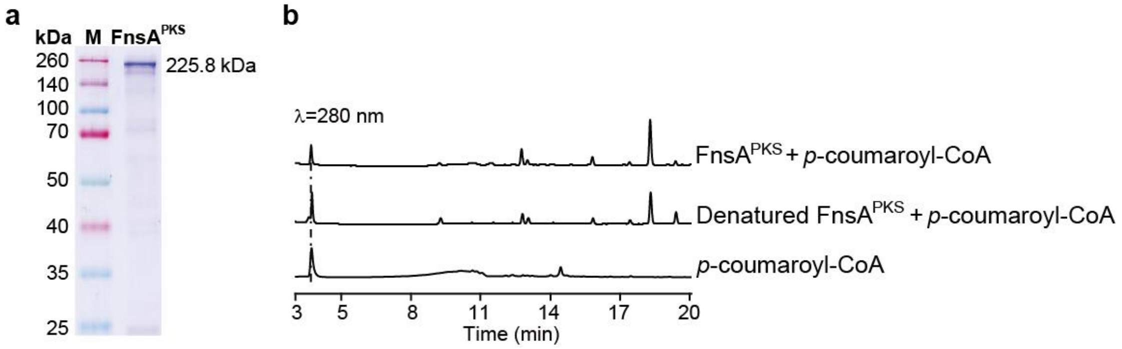

Supplementary Figure 7. *In vitro* enzymatic assay of FnsA$^{\mathbf{PKS}}$. a, SDS-PAGE analysis of purified FnsA^{PKS}. FnsA^{PKS} was expressed in *S. cerevisiae* and purified with *His*$_6$-tag. The yield of FnsA^{PKS} was 1 mg·L^{-1}. M, marker. **b**, HPLC analysis of the enzyme reactions of FnsA^{PKS} with *p*-coumaroyl-CoA. Each experiment (**a**) was repeated twice independently with similar results. Source data are provided as a Source Data file.

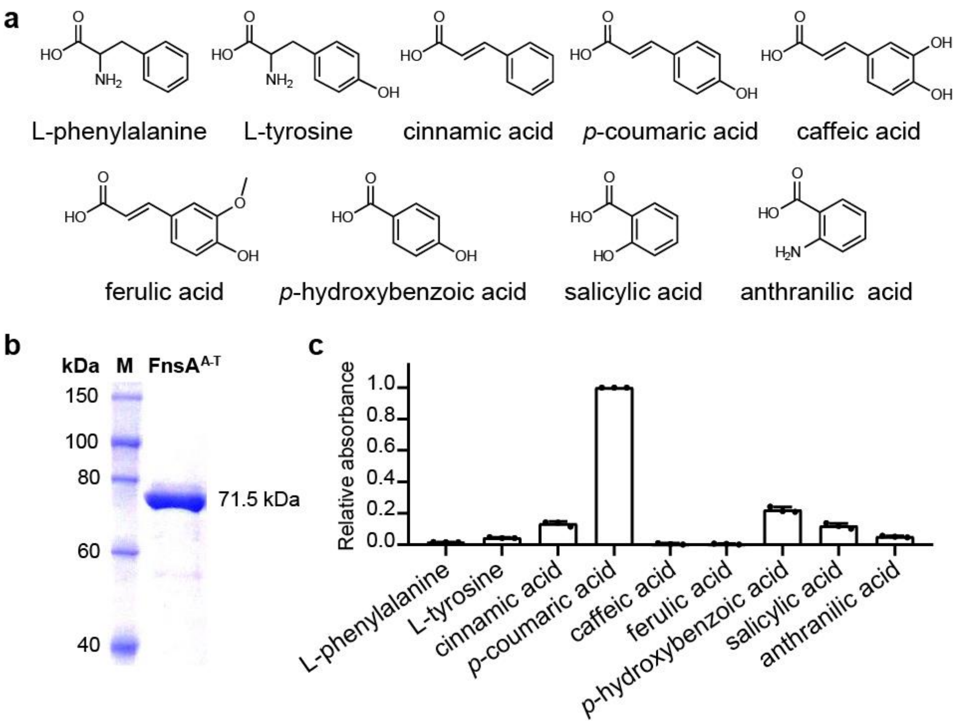

Supplementary Figure 8. Substrate specificity of the FnsA A domain. a, Structures of the tested substrates. **b**, SDS-PAGE of purified $FnsA^{A\text{-}T}$. $FnsA^{A\text{-}T}$ was expressed in *E. coli* and purified with *His*$_6$-tag. The yield of $FnsA^{A\text{-}T}$ was 15 $mg\cdot L^{-1}$. M, marker. **c**, Substrate profiling of the A domain in FnsA. All data represents the mean of n = 3 biologically independent samples and error bars show standard deviation. Each experiment (**b**) was repeated twice independently with similar results. Source data are provided as a Source Data file.

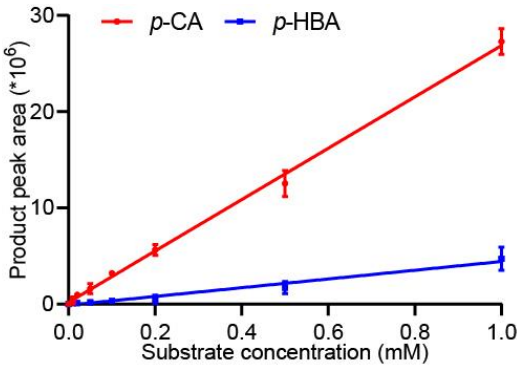

Supplementary Figure 9. Comparison of product yields toward 3 and 4 by feeding experiments in *S. cerevisiae* carrying *fnsA*. All data represents the mean of $n = 3$ biologically independent samples and error bars show standard deviation. Source data are provided as a Source Data file.

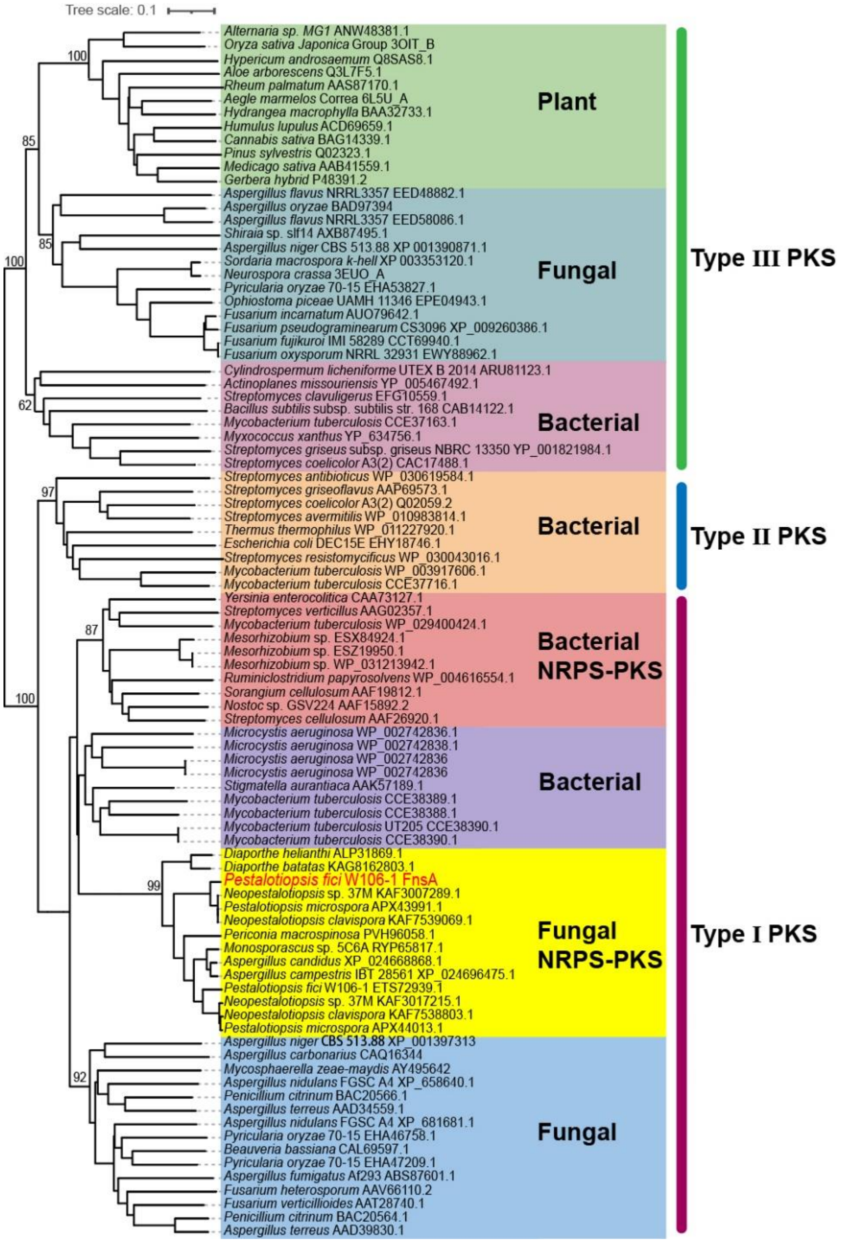

Supplementary Figure 10. Phylogenetic analysis of the FnsA KS domain. Scale bar, 0.1 substitutions per site. The FnsA KS domain is represented in red. The KS domains of FnsA homologues are indicated with yellow. The KS domains were extracted using a PKS-NRPS analysis website (at http://nrps.igs.umaryland.edu/nrps/). GenBank accession number of each protein is included.

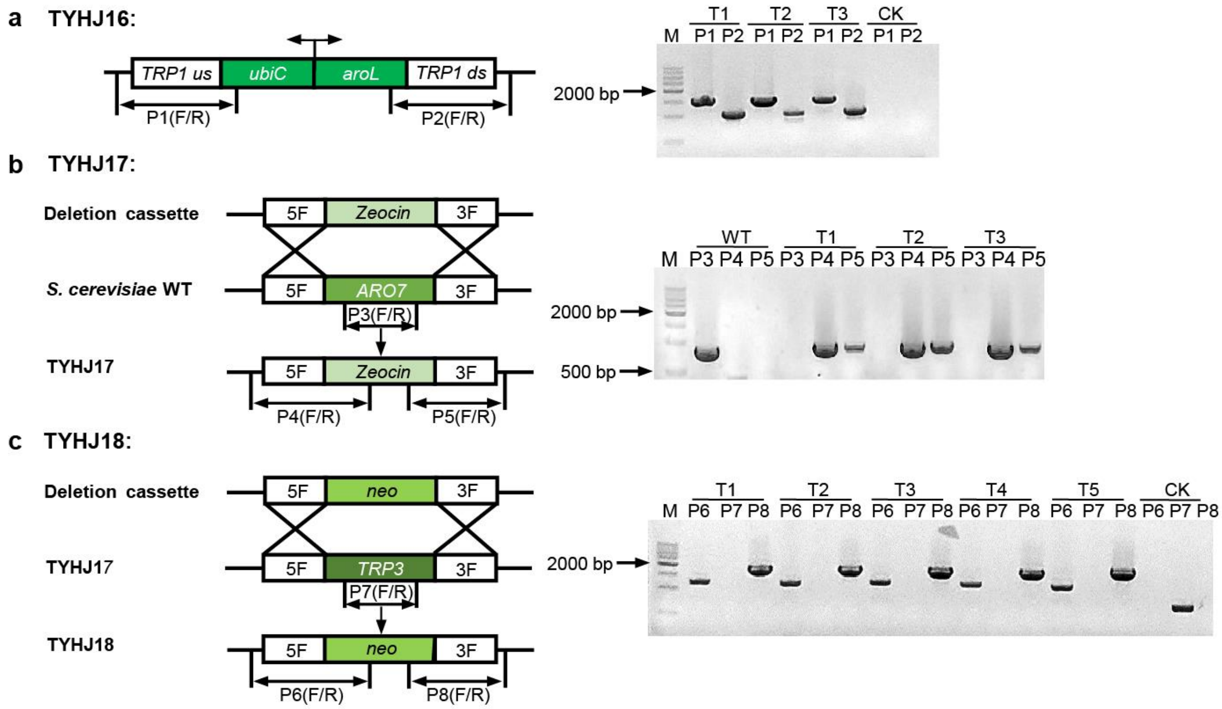

Supplementary Figure 11. Integration of *p*-hydroxybenzoic acid pathway and gene deletion of *ARO7* and *TRP3* in *S. cerevisiae* BJ5464-NpgA. a, Scheme illustration of the construction strategies of strain TYHJ16 and confirmation of TYHJ16 by diagnostic PCR. The specific bands (about 1.5 kb and 1.0 kb) were detected using the P1(F/R) and P2(F/R) primers in TYHJ16 (T1-T3) but not in CK. T, transformants. **b**, Scheme illustrations of the construction strategies of TYHJ17 and confirmation of TYHJ17 by diagnostic PCR. The specific bands (about 0.8 kb and 1.0 kb) were detected using the P4(F/R) and P5(F/R) primers in TYHJ17 (T1-T3) but not in CK. T, transformants. **c**, Scheme illustrations of the construction strategies of TYHJ18 and confirmation of TYHJ18 by diagnostic PCR. The specific bands (about 1.2 kb and 1.6 kb) were detected using the P6(F/R) and P8(F/R) primers in TYHJ18 (T1-T5) but not in TYHJ17. T, transformants. Each experiment (**a**-**c**) was repeated twice independently with similar results. Source data are provided as a Source Data file.

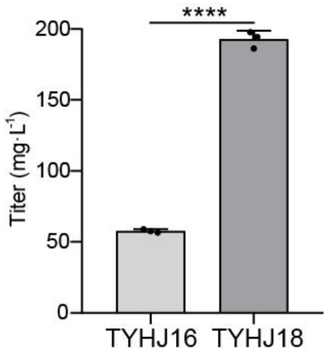

Supplementary Figure 12. The production yield of *p*-hydroxybenzoic acid in *S. cerevisiae* BJ5464-NpgA. Strains were grown in YPD medium. Cultures were sampled for metabolite detection after 96 h of growth. Statistical analysis was performed by using *t* test (two-tailed; ****p < 0.0001). All data represents the mean of n = 3 biologically independent samples and error bars show standard deviation. Source data are provided as a Source Data file.

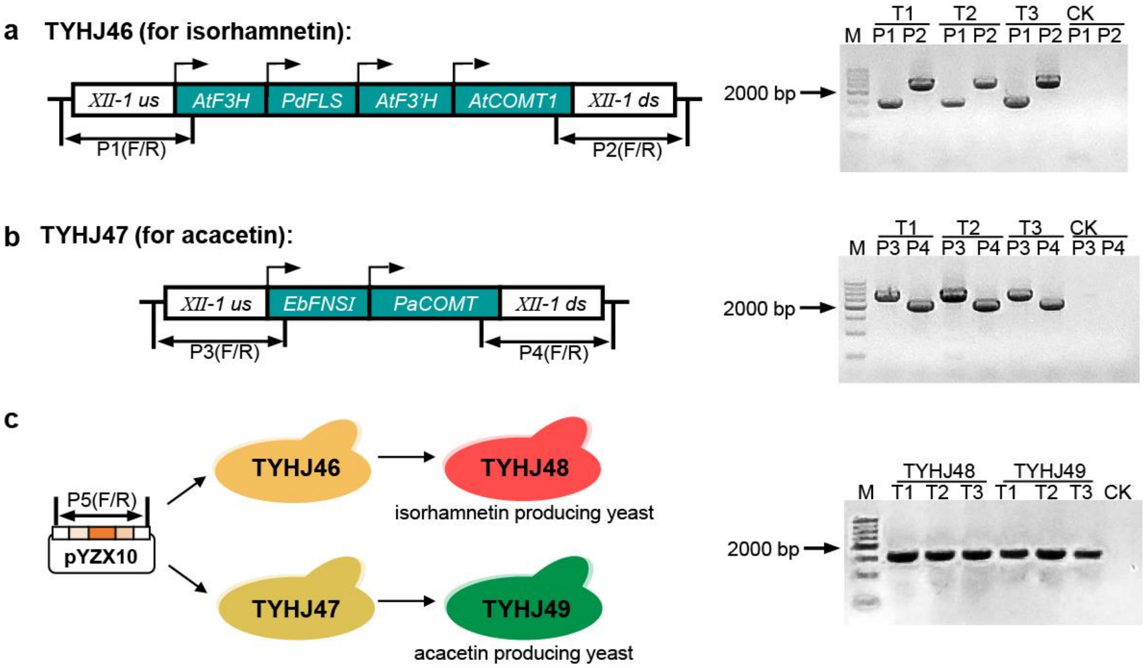

Supplementary Figure 13. Construction of isorhamnetin pathway and acacetin pathway in QL35. a, Scheme illustration of the construction of isorhamnetin pathway in QL35. The specific bands (about 1.5 kb and 2.5 kb) were detected using the P1(F/R) and P2(F/R) primers in TYHJ46 (T1-T3) but not in CK. T, transformants. **b**, Scheme illustration of the construction of acacetin pathway in QL35. The specific bands (about 2.5 kb and 2.0 kb) were detected using the P3(F/R) and P4(F/R) primers in TYHJ47 (T1-T3) but not in CK. T, transformants. **c**, Scheme illustration of the construction of TYHJ48 (isorhamnetin producing yeast) and TYHJ49 (acacetin producing yeast). The specific bands (about 1.5 kb) were detected using the P5(F/R) in TYHJ48 (T1-T3) and TYHJ49 (T1-T3) but not in CK. T, transformants. Each experiment (**a**-**c**) was repeated twice independently with similar results. Source data are provided as a Source Data file.

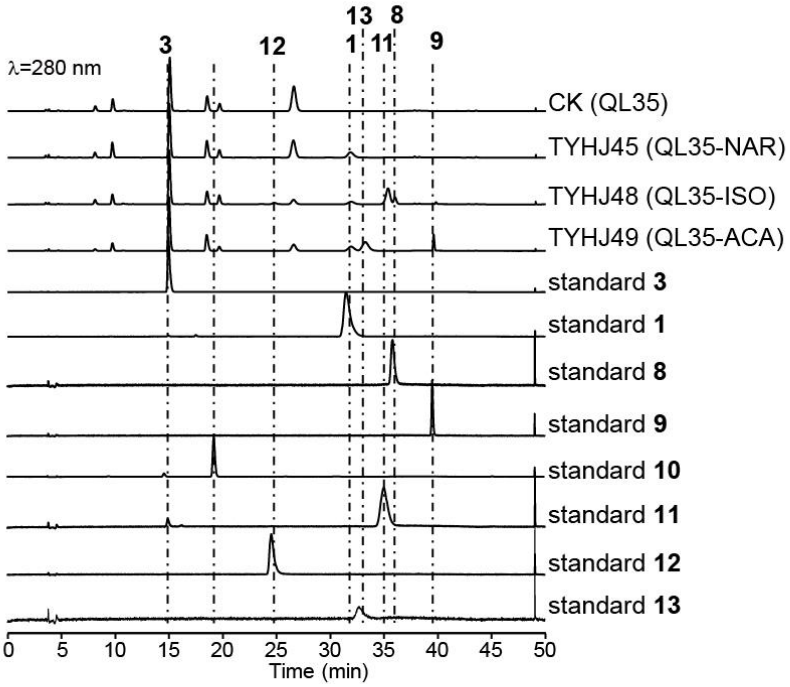

Supplementary Figure 14. HPLC analysis of the products from TYHJ45 (1-producing strain), TYHJ48 (8-producing strain) and TYHJ49 (9-producing strain). 10 was transformed into **11** completely in TYHJ48. **1**, naringenin; **3**, *p*-coumaric acid; **8**, isorhamnetin; **9**, acacetin; **10**, dihydrokaempferol; **11**, kaempferol; **12**, quercetin; **13**, apigenin.

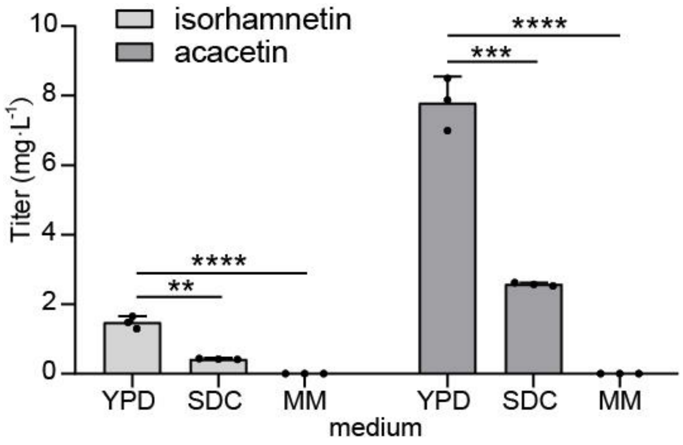

Supplementary Figure 15. The production of isorhamnetin (8) and acacetin (9) on three different media. Cultures from YPD, SDC and MM medium were sampled for metabolite detection after 96 h of growth. The production of **8** and **9** from SDC medium achieved 0.42 mg·L^{-1} and 2.6 mg·L^{-1}, respectively. No accumulation of **8** and **9** was detected in MM medium. Statistical analysis was performed by using One-way ANOVA (**$p = 0.005$, ***$p = 0.0003$, ****$p < 0.0001$). All data represents the mean of $n = 3$ biologically independent samples and error bars show standard deviation. Source data are provided as a Source Data file.

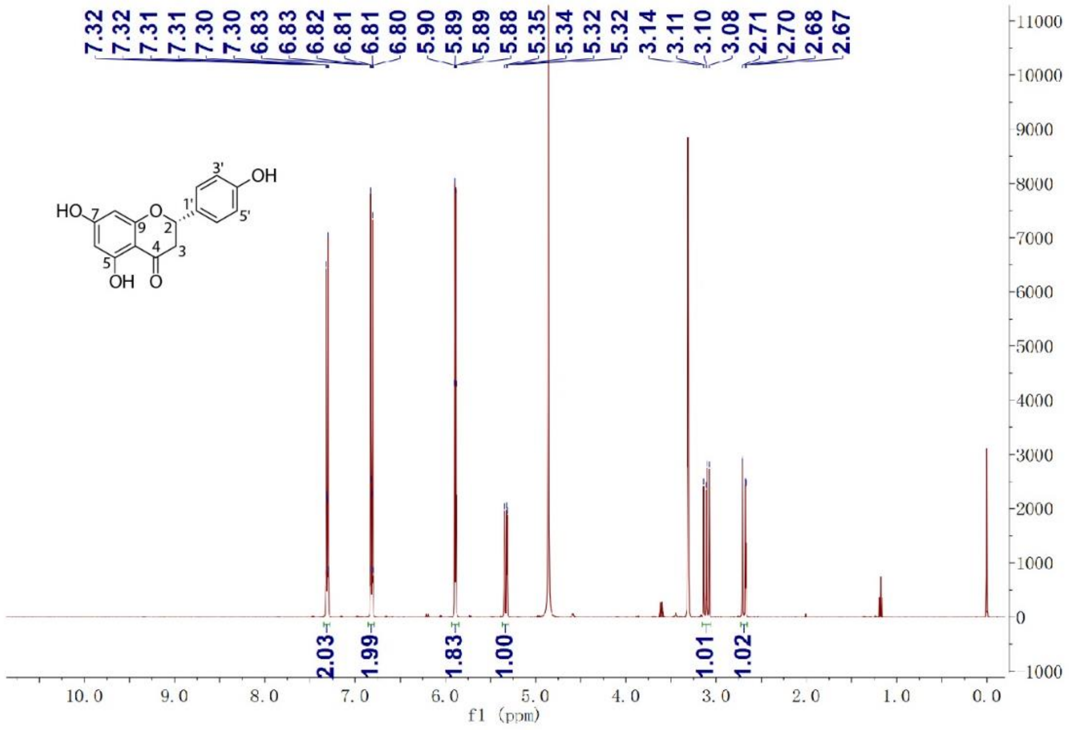

Supplementary Figure 16. ^{1}H NMR spectrum of compound 1 in Methanol-*d*$_4$ (500 MHz).

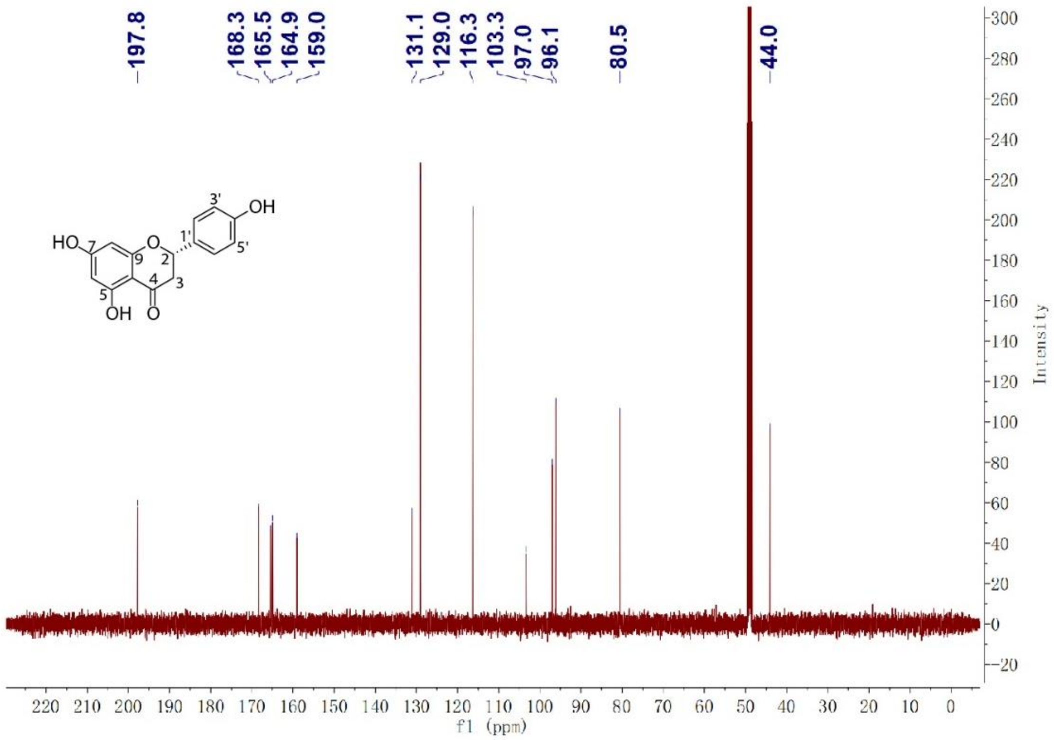

Supplementary Figure 17. ^{13}C NMR spectrum of compound 1 in Methanol-d_4 (125 MHz).

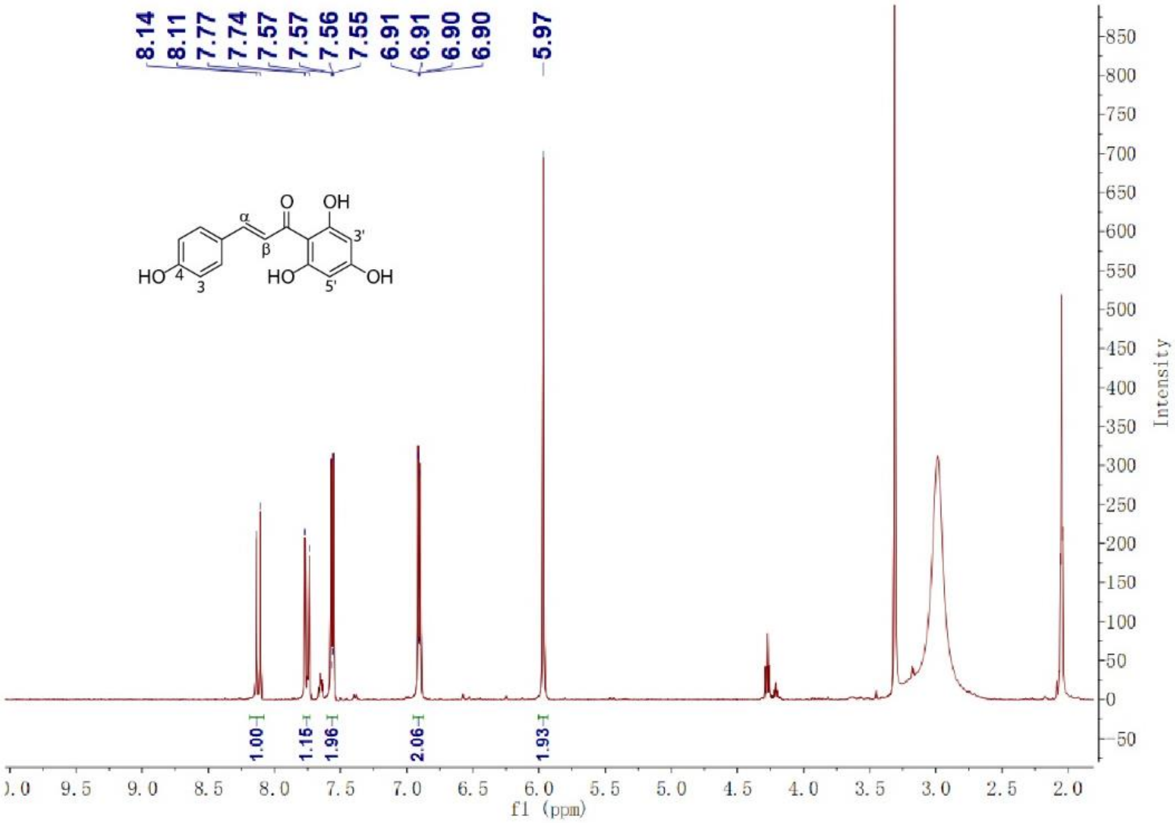

Supplementary Figure 18. ^{1}H NMR spectrum of compound 2 in Acetone-d_6 (500 MHz).

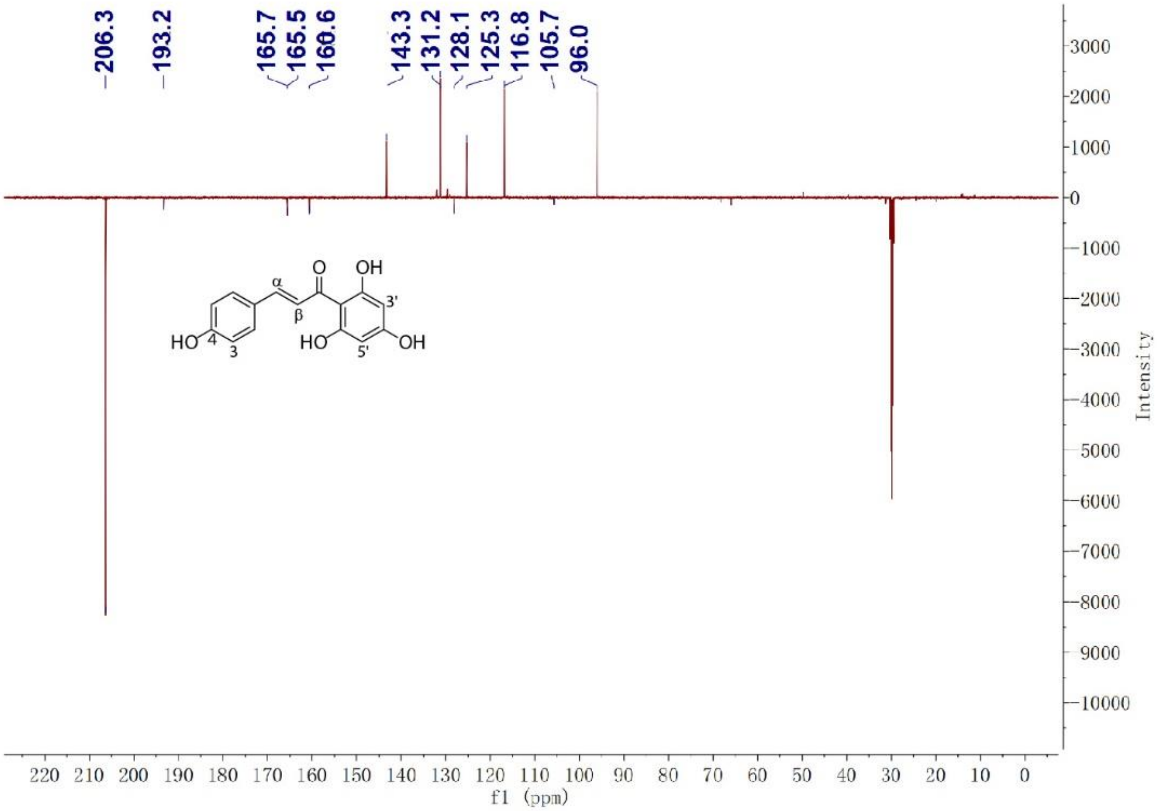

Supplementary Figure 19. ^{13}C NMR spectrum of compound 2 in Acetone-*d*$_6$ (125 MHz).

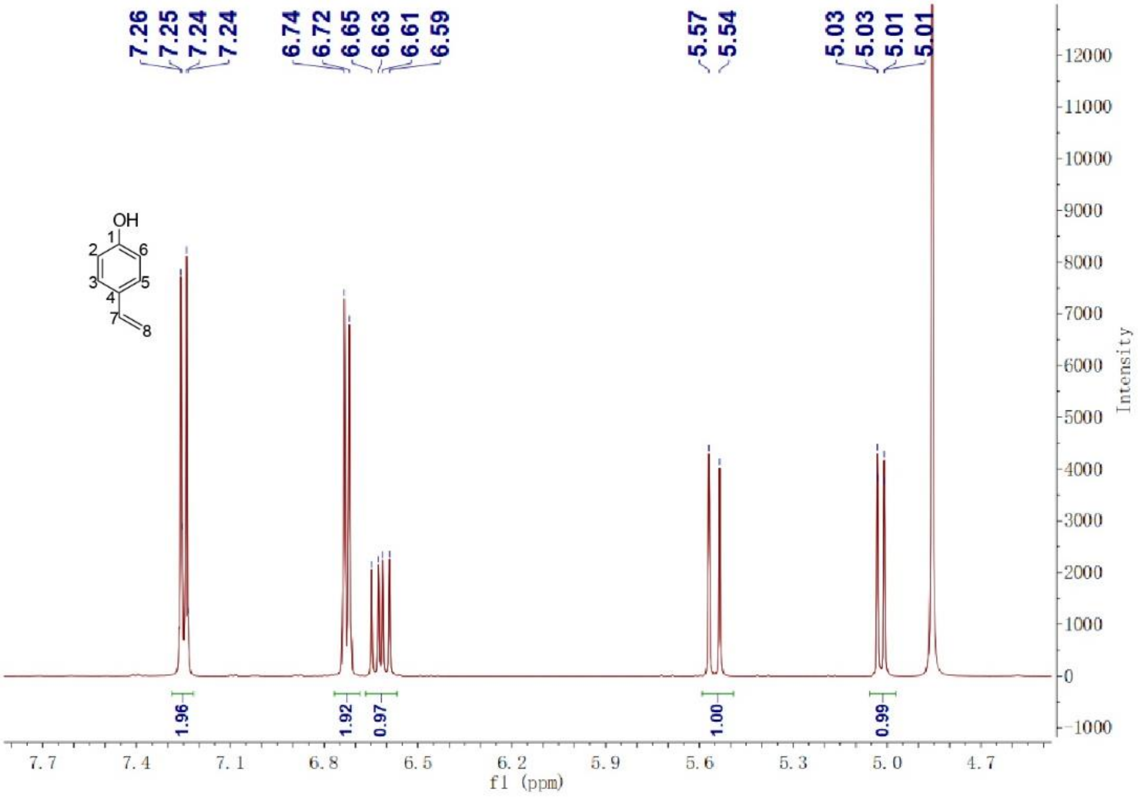

Supplementary Figure 20. ^{1}H NMR spectrum of compound 5 in Methanol-d_4 (500 MHz).

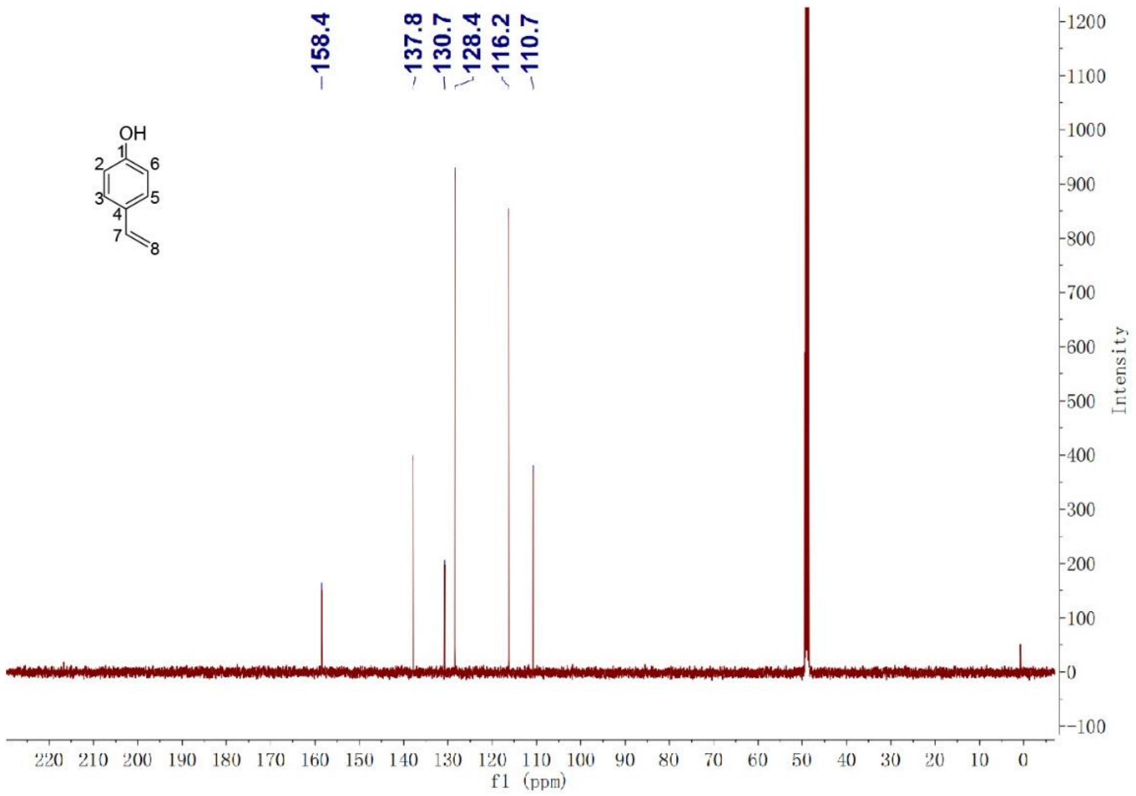

Supplementary Figure 21. ^{13}C NMR spectrum of compound 5 in Methanol-d_4 (125 MHz).

Supplementary Table 1. Functional annotations of *fnsA* surrounding genes.

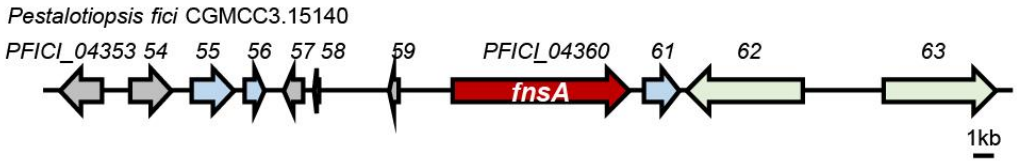

**Gene ID**	**Putative function**	**Organism**	**Identity (%)/ coverage (%)**
PFICI_04353	hypothetical protein	-	-
PFICI_04354	heterokaryon incompatibility protein-domain-containing protein	*Microdochium bolleyi*	48.85/ 100
PFICI_04355	glycoside hydrolase superfamily	*Pestalotiopsis* sp. NC0098	89.74/ 100
PFICI_04356	endo-alpha-1,5-arabinanase	*Pestalotiopsis* sp. NC0098	80.22/ 93
PFICI_04357	alpha/beta hydrolase fold-1	*Pestalotiopsis* sp. NC0098	53.41/ 98
PFICI_04358	hypothetical protein	-	-
PFICI_04359	mRNA' 3 end-processing YTH1	*Fusarium globosum*	45.14/ 88
PFICI_04360	hybrid NRPS-PKS	*Pestalotiopsis microspora*	90.29/ 99
PFICI_04361	glycoside hydrolase superfamily	*Pestalotiopsis* sp. NC0098	91.83/ 100
PFICI_04362	chitin synthase	*Pestalotiopsis* sp. NC0098	94.70/ 100
PFICI_04363	chitin synthase	*Pestalotiopsis* sp. NC0098	93.73/ 100

Supplementary Table 2. Genes involved in the biosynthesis of isorhamnetin and acacetin.

Gene	Function	Source	GenBank accession	Reference
ubiC	chorismate lyase	*Escherichia coli*	No. ECK4031	[2]
aroL	shikimate kinase	*Escherichia coli*	No. ECK0383	[2]
ARO7	chorismate mutase	*Saccharomyces cerevisiae*	No. 856173	[2]
TRP3	indole-3-glycerol-phosphate synthase	*Saccharomyces cerevisiae*	No. 853669	[2]
AtF3H	flavanone 3-hydroxylase	*Arabidopsis thaliana*	No. AY116957	[3]
AtF3'H	flavonol 3'-hydroxylase	*Arabidopsis thaliana*	No. AF271651	[4]
AtCOMT1	O-methyltransferase I	*Arabidopsis thaliana*	No. AY081565	[5]
PdFLS	flavonol synthase	*Populus deltoides*	No. TC74233*	[3]
EbFNSI	flavone synthase I	*Erigeron breviscapus*	No. AGK85254	[6]
PaCOMT	4'-O-methyltransferase	*Plagiochasma appendiculatum*	No. KY977687	[7]

* TIGR accession

Supplementary Table 3. The MS-MS mass spectrum data of *holo*-FnsA$^{A\text{-}T}$ binding with 3.

#	b^{+}	b^{2+}	b^{3+}	Seq.	y^{+}	y^{2+}	y^{3+}	#
1	164.0706	82.5389	55.3617	**Y**				20
2	235.1077	118.0575	79.0408	**A**	2476.1284	1238.5678	826.0476	19
3	350.1347	175.5710	117.3831	**D**	2405.0913	1203.0493	802.3686	18
4	479.1773	240.0923	160.3973	**E**	2290.0643	1145.5358	764.0263	17
5	566.2093	283.6083	189.4080	**S**	2161.0217	1081.0145	721.0121	16
6	713.2777	357.1425	238.4308	**F**	2073.9897	1037.4985	692.0014	15
7	800.3098	400.6585	267.4414	**S**	1926.9213	963.9643	642.9786	14
8	937.3687	469.1880	313.1277	**H**	1839.8892	920.4483	613.9679	13
9	1050.4527	525.7300	350.8224	**L**	1702.8303	851.9188	568.2816	12
10	1107.4742	554.2407	369.8296	**G**	1589.7462	795.3768	530.5869	11
11	1220.5583	610.7828	407.5243	**L**	1532.7248	766.8660	511.5798	10
12	1321.6060	661.3066	441.2068	**T**	1419.6407	710.3240	473.8851	9
13	1894.7600	947.8836	632.2582	**S**	1318.5930	659.8002	440.2025	8
14	2025.8005	1013.4039	675.9384	**M**	745.4390	373.2231	249.1512	7
15	2096.8376	1048.9225	699.6174	**A**	614.3985	307.7029	205.4710	6
16	2153.8591	1077.4332	718.6246	**G**	543.3613	272.1843	181.7920	5
17	2252.9275	1126.9674	751.6474	**V**	486.3399	243.6736	162.7848	4
18	2351.9959	1176.5016	784.6702	**V**	387.2715	194.1394	129.7620	3
19	2465.0800	1233.0436	822.3649	**L**	288.2030	144.6052	96.7392	2
20				**R**	175.1190	88.0631	59.0445	1

Note: The labels "b" and "y" assign the N- and C-terminal fragment ions of the peptide produced by collision-induced fragmentation at the peptide bond in the mass spectrometer. $^{1+}$, $^{2+}$, $^{3+}$ represent the numbers of the charge.

Supplementary Table 4. The MS-MS mass spectrum data of *holo*-FnsA$^{A\text{-}T}$ binding with 4.

#	b^+	b^{2+}	b^{3+}	Seq.	y^+	y^{2+}	y^{3+}	#
1	164.0706	82.5389	55.3617	**Y**				20
2	235.1077	118.0575	79.0408	**A**	2450.1127	1225.5600	817.3758	19
3	350.1347	175.5710	117.3831	**D**	2379.0756	1190.0414	793.6967	18
4	479.1773	240.0923	160.3973	**E**	2264.0487	1132.5280	755.3544	17
5	566.2093	283.6083	189.4080	**S**	2135.0061	1068.0067	712.3402	16
6	713.2777	357.1425	238.4308	**F**	2047.9740	1024.4907	683.3295	15
7	800.3098	400.6585	267.4414	**S**	1900.9056	950.9564	634.3067	14
8	937.3687	469.1880	313.1277	**H**	1813.8736	907.4404	605.2960	13
9	1050.4527	525.7300	350.8224	**L**	1676.8147	838.9110	559.6097	12
10	1107.4742	554.2407	369.8296	**G**	1563.7306	782.3689	521.9151	11
11	1220.5583	610.7828	407.5243	**L**	1506.7091	753.85820	502.9079	10
12	1321.6060	661.3066	441.2068	**T**	1393.6251	697.3162	465.2132	9
13	1852.7494	926.8784	618.2547	**S**	1292.5774	646.7923	431.5306	8
14	1999.7849	1000.3961	667.2665	**M-oxidation**	761.4339	381.2206	254.4828	7
15	2070.8220	1035.9146	690.9455	**A**	614.3985	307.7029	205.4710	6
16	2127.8434	1064.4254	709.9527	**G**	543.3613	272.1843	181.7920	5
17	2226.9119	1113.9596	742.9755	**V**	486.3399	243.6736	162.7848	4
18	2325.9803	1163.4938	775.9983	**V**	387.2715	194.1394	129.7620	3
19	2439.0644	1220.0358	813.6930	**L**	288.2030	144.6052	96.7392	2
20				**R**	175.1190	88.0631	59.0445	1

Note: The labels "b" and "y" assign the N- and C-terminal fragment ions of the peptide produced by collision-induced fragmentation at the peptide bond in the mass spectrometer. $^{1+}$, $^{2+}$, $^{3+}$ represent the numbers of the charge.

Supplementary references

1. Mukai, N., Masaki, K., Fujii, T., Kawamukai, M. & Iefuji, H. *PAD1* and *FDC1* are essential for the decarboxylation of phenylacrylic acids in *Saccharomyces cerevisiae*. *J. Biosci. Bioeng.* **109**, 564-569 (2010).
2. Averesch, N.J.H., Winter, G. & Krömer, J.O. Production of *para*-aminobenzoic acid from different carbon-sources in engineered *Saccharomyces cerevisiae*. *Microb. Cell. Fact.* **15**, 89 (2016).
3. Duan, L. *et al.* Biosynthesis and engineering of kaempferol in *Saccharomyces cerevisiae*. *Microb. Cell. Fact.* **16**, 165 (2017).
4. Marin, L., Gutierrez-Del-Rio, I., Entrialgo-Cadierno, R., Villar, C.J. & Lombo, F. *De novo* biosynthesis of myricetin, kaempferol and quercetin in *Streptomyces albus* and *Streptomyces coelicolor*. *PLoS. One.* **13**, e0207278 (2018).
5. Moinuddin, S.G. *et al.* Insights into lignin primary structure and deconstruction from *Arabidopsis thaliana* COMT (caffeic acid *O*-methyl transferase) mutant *Atomt1*. *Org. Biomol. Chem.* **8**, 3928-46 (2010).
6. Liu, X. *et al.* Engineering yeast for the production of breviscapine by genomic analysis and synthetic biology approaches. *Nat. Commun.* **9**, 448 (2018).
7. Wang, X., Shao, A., Li, Z., Policarpio, L. & Zhang, H. Constructing *E. coli* co-cultures for *de novo* biosynthesis of natural product acacetin. *Biotechnol. J.* **15**, e2000131 (2020).
